# The Feasibility of Choosing D4 Embryo Transfer—Analysis of Nanomaterials Affecting the Outcome of Frozen-Thaw Embryo Transfer

**DOI:** 10.1155/2022/1364865

**Published:** 2022-10-10

**Authors:** Chang Tan, Xiliang Wang, Lishuang Luo, Jinyan Zhang, Pengshu Zou, Wei Wei, Yuexin Yu

**Affiliations:** Department of Reproductive Medicine, General Hospital of Northern Theater Command, Shenyang 110003, Liaoning, China

## Abstract

FET is to resuscitate the endometrium and transfer the embryo into the uterus after the endometrium is ready. The quality of transferred embryos is an important factor affecting the outcome of assisted reproductive technology. This paper aims to explore the feasibility of D4 frozen-thaw embryo transfer and analysis of related factors affecting the outcome of freeze-thaw embryo transfer. A retrospective analysis of the clinical data of 2925 patients who received frozen-thaw embryo transfer (FET) in the Department of Reproductive Medicine, General Hospital of Northern Theater Command from January 1, 2017 to July 31, 2019. Including the woman's age, body mass index (BMI), endometrial thickness on the day of transplantation, number of embryos to be transferred, and type of embryos to be transferred. A single factor, multivariate logistic regression and nomogram were used to analyze the influence of different factors on the clinical outcome of FET. Nanomedicines and related nanomedicines are rapidly developing and establishing their importance in embryo transfer. This paper uses nanomaterials to explore the feasibility of D4 frozen-thawed embryo transfer. The woman's age, endometrial thickness on the day of transplantation, BMI, the number of embryos transferred, and the type of embryos transferred all affect the outcome of FET. The pregnancy rate of the D5 and D4 transplantation groups was, respectively, higher than that of the D3 transplantation group, with statistically significant differences. In the FET cycle, the age of the woman, endometrial thickness on the day of transplantation, the number of embryos transferred, and the type of embryos transferred are all independent factors influencing the outcome of FET. D5 blastocyst is the easiest to get pregnant, and that has the best clinical outcome which is better than the D6 blastocyst group; D4 morula and D5 blastocyst FET have little difference in clinical pregnancy outcomes, but both of them are significantly better than D3 cell embryos, so D4 morula can be considered for transplantation in the FET cycle. In conclusion, whether it is a patient who has failed the fresh cycle transplantation or the whole embryo freezing cycle whose transplantation is canceled due to high hormone levels on the transplantation day, FET is required.

## 1. Introduction

With the rapid development of assisted reproductive technology, the technology of embryo cryopreservation and embryo culture technique in vitro are becoming more and more perfect and stable, and they are widely used by reproductive centers around the world. On the one hand, frozen-thawed embryo transfer (FET) can reasonably control the number of embryos transferred and avoid the occurrence of multiple pregnancies. On the other hand, FET can not only reduce the occurrence of ovarian hyperstimulation syndrome when the embryo is synchronized with the endometrial “implantation window,” but it can also increase the embryo implantation rate and the cumulative pregnancy rate of a single oocyte retrieval cycle, and reduce the patients' treatment costs [1, 2]. FET has been widely used clinically. Many factors affect the clinical outcome of FET, and embryo quality is one of them. Many research results suggest that blastocyst should be thawed first in the cycle of FET, but in actual clinical work, due to individual differences in patients, the success rate of each blastocyst transfer cannot be guaranteed. There are many studies on the influence of the endometrial preparation program on FET pregnancy, but most studies believe that it has no effect [3, 4]. Studies have reported that under natural physiological conditions, early embryos develop in the fallopian tube and do not enter the uterine cavity until they are close to the blastocyst stage [5]. Blastocyst transfer is more in line with natural physiological phenomena, so blastocyst transfer can improve the clinical pregnancy rate and embryo implantation rate [6–8]. There have always been different research reports on the developmental potential of blastocysts at different times. Some scholars believe that there is no significant difference in clinical pregnancy rates after transplantation of D5 and D6 freeze-thawed blastocysts [9, 10]. Studies have also shown that the clinical pregnancy rate of D5 freeze-thawed blastocyst transplantation is significantly higher than that of D6 [11].

Therefore, how to choose the most suitable embryo for transplantation to increase the pregnancy rate while minimizing the number of the embryo and the multiple pregnancy rate in the cycle of FET is an urgent problem to be solved in clinical work. This article retrospectively analyzes the clinical outcomes of 2925 FET cycles from January 2017 to July 2019 and further compares frozen-thawed embryos at different times and other related factors that affect the outcome of FET, providing a useful reference for the selection of embryos for FET cycles.

## 2. Materials and Methods

### 2.1. Research Object

The data of patients who underwent FET in our department from 2017.01 to 2019.07 were collected, and a total of 2925 cycles were included for retrospective analysis. Inclusion criteria are as follows: (1) Age <45 years old (2) Number of embryo transfers ≤2, excluding (1) patients with previous ovarian surgery history, genital malformations, endometriosis, submucosal fibroids and intrauterine adhesions, hydrosalpinx and other factors that may affect embryo implantation. (2) Chromosomal abnormality in either partner (3) cycles of preimplantation genetic testing (PGT). The endometrium preparation of all patients in this study adopted hormone replacement therapy. The study was reviewed and approved by the medical Ethics Committee of our hospital, and the freeze-thaw embryo transfer was performed with the full informed consent of the patients. Collecting patients data, patients are divided into ≤35 years old group, >35 years old group by age; that are divided into ≤0.8 cm group, 0.81∼1.0 cm group, and >1.0 cm group according to endometrial thickness on the embryo transfer day; and divided into ≤18.5 group, 18.6∼23.9 group, 24∼26.9 group, 27∼29.9 group, and >30 groups by BMI; the number of embryos transferred is divided into 1 embryo transfer group and 2 embryos group; according to the morphology of embryo transfer, they are divided into D3 frozen-thawed embryo transfer group, D4 frozen-thawed embryo transfer group, D5 frozen-thawed embryo transfer group, and D6 frozen-thawed embryo transfer group.

### 2.2. Research Methods

Among assisted reproductive technology, such as perform in vitro fertilization (IVF) or intracytoplasmic sperm injection (ICSI). The first day after fertilization, embryologists judge fertilization by the presence of double pronucleus, and record the number of blastomeres and embryo grading according to the Peter cleavage scoring system. They select D3 high-quality cleavage embryos to freeze, and the remaining embryos continue to be cultured after the patients' informed consent. D4 embryo develops into morula embryos. If the D5 or D6 embryo develops into blastocyst that formation would be recorded, using Gardner scoring system scores blastocysts [12].

#### 2.2.1. Embryo Culture and Score

The standard of embryo freezing in this laboratory is: on the D3, we select high-quality cleavage embryos that divide into 7–9 cells, with uniform blastomeres and fragment rates less than 10% for freezing [13]. If the embryo developed into a morula on D4, that would be frozen. The blastocysts would be frozen with a score of 3BC or above on D5. If D5 does not develop blastocysts that meet the standard, continue to culture until day 7, during which embryos that meet the standard will be frozen again. When the patient has no blastocysts that meet the standards of 3BC or 3CB, the secondary embryos will be frozen after being informed and signed informed consent.

#### 2.2.2. Cryopreservation

We adopt vitrification freezing technology, before freezing, laser drilling is used to artificially shrink the blastocyst to release the liquid in the blastocyst cavity. The resuscitated embryos are incubated with laser. For the blastocyst and morula, the zona pellucida away from the inner cell mass is punched through, and the zona pellucida of cleavage embryo is thinned. The resuscitated embryos are transferred to the pre-equilibrated culture medium the previous day, and placed in the incubator for culture. They are observed before transplantation, the survival of embryos is determined according to whether the blastocyst cavity, morulae are re-expanded, and the number of cells survived by cleavage embryos after thawing [14].

#### 2.2.3. Endometrial Preparation

Compared with fresh cycle transplantation, the FET cycle endometrial and embryonic development are more synchronized. There are many studies on the influence of the endometrial preparation program on FET pregnancy, but most studies believe that it has no effect [3, 4]. All patients enrolled in this study used a hormone replacement cycle: starting from the 3rd to 5th day of the menstrual cycle, oral estradiol valerate (Progynat, Bayer, Germany) was administered 4–6 mg daily, and the thickness of endometrium was monitored by ultrasound after 7 days. Thickness and hormone levels. Adjust the dosage of estradiol valerate in time. When the endometrial thickness needs to reach at least 0.6 cm, an intramuscular injection of 40–60 mg/d of progesterone is injected to support the corpus luteum. Blastocyst transplantation was performed on the 5th or 6th day after the ketone injection. D5 or D6 blastocysts were selected for resuscitation and transplantation.

#### 2.2.4. Observation Indicators and Evaluation Criteria

Test blood human chorionic gonadotropin (*β*-HCG) on the 14th day after embryo transfer. If it is positive and 4 to 5 weeks after transplantation, a vaginal B-ultrasound examination is performed. The gestational sac and heart tube pulsation in the uterus are clinically pregnant, and the corpus luteum support is continued until 10 to 12 weeks of pregnancy, and the follow-up is to live birth.

### 2.3. Statistical Methods

Statistical analysis was performed using SPSS 25.0 software. Continuous variable data is expressed in the form of mean ± standard deviation *x* ± *s*, independent samples *t*-test is used, and *t*' test is used when the variance is not homogeneous; categorical variable data is expressed in the form of *n* (%), and the nonparametric chi-square test is used for group analysis. If the frequency is less than 5 and greater than or equal to 1, the continuous correction chi-square test is used; logistic regression is used for multivariate regression analysis; *R* language (4.1.1) is used to draw a nomogram. The analysis results were considered statistically significant at *P* ≤ 0.05.

## 3. Results

### 3.1. Basic Data of Patients during the Frozen-Thaw Embryo Transfer Cycle

The basic clinical data of 2925 patients were collected, with an average age of 33.08 years, an average endometrial thickness of 0.98 cm on the day of transfer, and an average BMI of 23.44. The specific distribution of the number of transferred embryos and the types of transferred embryos is shown in Table 1.

### 3.2. Analysis of Single Factors Affecting the Pregnancy Outcome of Frozen-Thaw Embryo Transfer Cycle

Univariate analysis of factors that may affect the outcome of FET in patients showed that the woman's age, endometrial thickness, BMI, number of embryos transferred, and embryos on the day of transfer all affected the clinical outcome of FET, and the differences were statistically significant (*P* < 0.05), see Table 2 for details.

### 3.3. Analysis of Multiple Factors Affecting the Pregnancy Outcome of Frozen-Thaw Embryo Transfer Cycle

Multivariate logistic regression analysis showed (Hosmer–Lemeshaw test *P*=0.897), the woman's age, endometrial thickness on the day of transfer, BMI, and the embryo on the day of transfer were the influencing factors of pregnancy in the FET cycle; Hosmer–Lemeshaw test (*P*=0.505). Among them, the clinical outcome of FET of D4 frozen-thawed embryos was better than that of D3 frozen-thawed embryos, and the difference was statistically significant (*P* < 0.05), see Table 3 for details.

### 3.4. Multivariate Analysis of Transferring D4 Frozen-Thawed Embryos and D5 Frozen-Thawed Embryos as Controls

Using D4 frozen-thawed embryos as the control standard, multivariate logistic regression analysis showed that in the FET cycle, the clinical outcome of D4 frozen-thawed embryos was better than that of D3 frozen-thawed embryos, and the difference was statistically significant (*P* < 0.05), but D4 frozen-thawed embryos had a statistically significant difference (*P* < 0.05). There was no significant difference in the outcome of frozen-thawed embryos with D5 and D6 (*P* > 0.05), see Table 4 for details.

Taking D5 frozen-thawed embryos as the control standard, multivariate logistic regression analysis showed that in the FET cycle, the clinical outcome of D5 frozen-thawed embryos was better than that of D6 and D3 frozen-thawed embryos, and the difference was statistically significant (*P* < 0.05). There was no significant difference in the outcomes of thawed embryos and D4 frozen-thawed embryos (*P* > 0.05), see Table 5 for details.

### 3.5. Nomograms of Factors Influencing the Outcome of Frozen-Thaw Embryo Transfer Cycle

**Figure 1:**
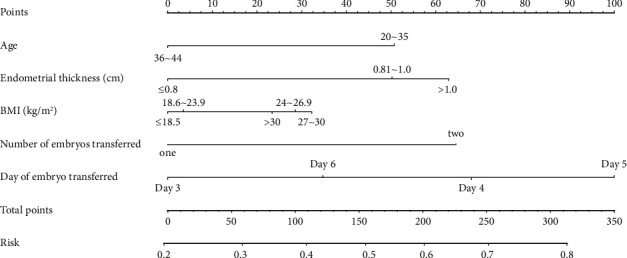
Nomograms of factors influencing the outcome of frozen-thaw embryo transfer cycle.

The nomogram (C-Index = 0.628) intuitively shows the correlation between the woman's age, endometrial thickness, body mass index BMI, the number of embryos transferred and the embryos on the first day of transfer and other factors with FET pregnancy, among which age is less than or equal to 35 age, endometrial thickness on the day of transfer >1.0 cm, BMI greater than or equal to 27 and less than or equal to 29.9, and it is relatively easier to get pregnant after transferring 2 embryos, D5 frozen-thawed embryos have the best correlation with pregnancy, followed by D4 frozen-thawed embryos, and D3 frozen-thawed embryos. Embryo pregnancy had the worst correlation and was relatively least fertile. See Figure 1 for details.

## 4. Discussion

FET is to resuscitate the endometrium and transfer the embryo into the uterus after the endometrium is ready, so that the endometrium and embryo development tend to be synchronized, the time interval is shortened between further development and implantation after embryo transfer, and the occurrence of complications such as ectopic pregnancy is reduced [15, 16].

As we all know, the quality of transferred embryos is an important factor affecting the outcome of assisted reproductive technology. The study in this article shows that D5 freeze-thawed blastocysts are the most likely to become pregnant in the FET cycle, and the clinical outcome is significantly better than D6 freeze-thawed blastocysts and D3 freeze-thawed blastocysts. The difference is statistically significant, which confirms the second point of view. The reason may be that the development of D6 blastocysts lags one day to form blastocysts. Under the condition that the embryos are recovered without damage, it may be that their developmental speed affects their developmental potential and embryo quality.

There are few studies on D4 freeze-thaw mulberry embryo transfer. Embryo development is a “survival of the fittest” selection process. Fertilized eggs gradually cleavage and develop into cellular embryos, morulae, and blastocysts. The morula is an embryo morphology between the cellular embryo and the blastocyst. The embryonic cells at the morula stage are in a dense or semidense state. Some cells that develop faster will also form early blastocysts, and the cells are tightly connected to form a network. The structure, which constitutes the embryonic skeleton, has good resistance to some drastic changes in the mother's body, avoids adverse reactions such as miscarriage, and thus increases the pregnancy rate [17]. Studies have shown that even high-quality embryos at the cleavage stage maybe 60% aneuploid, and the aneuploidy rate of embryos that develop into morula and blastocysts can be reduced to about 30% [18]. On the one hand, mulberry embryos are developed from cleavage stage embryos with good developmental potential. Embryo culture is a natural selection process for the survival of the fittest. Mulberry embryos are the elimination of developmentally retarded and unfused embryos and form semifusion or fusion. Potential embryo. On the other hand, morula freezing is better than blastocyst freezing. Blastocyst freezing requires artificial shrinkage to remove a large amount of water in the blastocyst cavity. It is easy to form ice crystals and cause embryo damage, and insufficient dehydration affects the thawing effect [19–23], artificial shrinkage also increases embryo manipulation and in vitro exposure time, which may affect embryo safety. In addition, blastocyst culture requires embryos to be cultured for 5–6 days, which prolongs the exposure time of embryos in vitro. Cell quality and culture environment may affect the development process of embryos, and even affect the formation of blastocysts, resulting in no embryos available. Risk [24].

## 5. Conclusion

To sum up, whether it is a patient who has failed the fresh cycle transplantation or the whole embryo freezing cycle whose transplantation is canceled due to high hormone levels on the transplantation day, FET is required. In the FET cycle, the patient's age, BMI, and embryo development should be fully considered. According to the actual conditions and blastocyst formation, the transplantation plan is tailored for each patient, the appropriate endometrial thickness is adjusted, and the embryos with good development potential are selected for transfer. In the case of patients with more blastocysts, it is recommended to give priority to the recovery of D5 freeze-thawed blastocysts.

The results of this study show that the FET outcome of D5 freeze-thawed blastocysts is the most relevant to pregnancy; if D5 does not form blastocysts, D6 blastocysts can be selected; but if the patient's blastocyst quality is poor or there is no blastocyst formation or the experience of blastocyst transfer failure, D4 freeze-thawed morulae can be considered for FET; this study found that D4 freeze-thaw mulberry embryos and D5 freeze-thawed blastocysts have a similar pregnancy probability There is no significant difference in clinical outcome, and it is significantly better than D3 freeze-thaw cleavage embryo. Freezing and thawing mulberry embryos for FET is a viable option.

However, there are few studies on D4 freeze-thaw mulberry embryo transfer. And the data are limited in our study. In the future work, we need to collect more data to obtain more scientific results. If some patients have special conditions or have no transplantable D5 blastocysts, using D4 morula for FET is also a new and feasible option, which not only increases FETs. The flexibility of FET, and provides a new idea for the selection of FET embryos.

## Figures and Tables

**Table 1:** Basic data of patients during the frozen-thaw embryo transfer cycle.

	N (%)	$\bar{x}$ *±* *s*
Age	2925	33.08 ± 4.14
≤35	2154 (73.6)	31.20 ± 2.83
>35	771 (26.4)	38.34 ± 2.25

Endometrial thickness (cm)	2925	0.98 ± 0.21
≤0.8	688 (23.5)	0.72 ± 0.078
0.81∼1.0	1247 (42.6)	0.94 ± 0.061
>1.0	990 (33.8)	1.21 ± 0.13

BMI (kg/m2)	2925	23.44 ± 3.70
≤18.5	175 (5.9%)	17.75 ± 0.83
18.6∼23.9	1573 (53.7%)	21.32 ± 1.46
24∼26.9	669 (22.8%)	25.29 ± 0.83
27∼29.9	331 (11.3%)	28.18 ± 0.82
>30	177 (6.0%)	31.99 ± 1.80

Number of embryos transferred		
1	726 (24.8)	
2	2199 (75.1)	

Day of embryo transferred		
D3	1234 (42.1)	
D4	53 (1.8)	
D5	725 (24.7)	
D6	913 (31.2)	

**Table 2:** Analysis of single factors affecting the pregnancy outcome of frozen-thaw embryo transfer cycle.

	No pregnancy	Pregnancy	*t/x2*	*P*
Age				
$\bar{x}$ *±* *s*	33.80 ± 4.35	32.50 ± 3.86	8.48	*P* < 0.001
≤35	890 (67.58)	1264 (78.61)	45.37	*P* < 0.001
>35	427 (32.42)	344 (21.39)		

Endometrial thickness (cm)				
$\bar{x}$ ± *s*	0.96 ± 0.22	0.93 ± 0.2	−4.80	*P* < 0.001
≤0.8	372 (28.25)	316 (19.65)	31.65	*P* < 0.001
0.81∼1.0	543 (41.23)	704 (43.78)		
>1.0	402 (30.52)	588 (36.57)		

BMI (kg/m2)				
$\bar{x}$ ± *s*	23.22 ± 3.61	23.61 ± 3.76	−2.83	*P*=0.005
≤18.5	84 (6.38)	91 (5.7)	10.18	*P*=0.037
18.6∼23.9	744 (56.49)	829 (51.55)		
24∼26.9	282 (21.40)	387 (24.1)		
27∼29.9	132 (10.00)	199 (12.4)		
>30	75 (5.70)	102 (6.3)		

Number of embryos transferred				
1	369 (28.02)	357 (22.20)	13.13	*P* < 0.001
2	948 (71.98)	1251 (77.80)		

Day of embryo transferred				
D3	616 (46.77)	618 (38.43)	44.25	*P* < 0.001
D4	19 (1.44)	34 (2.11)		
D5	254 (19.29)	471 (29.29)		
D6	428 (32.50)	485 (30.16)		

**Table 3:** Analysis of multiple factors affecting the pregnancy outcome of frozen-thaw embryo transfer cycle.

	OR (95% CI)	*P*
Age		*P* < 0.001
≤35		
>35	0.64 (0.54–0.76)	*P* < 0.001

Endometrial thickness (cm)		*P* < 0.001
≤0.8		
0.81∼1.0	1.56 (1.28–1.89)	*P* < 0.001
>1.0	1.74 (1.42–2.13)	*P* < 0.001

BMI (kg/m2)		*P*=0.74
≤18.5		
18.6∼23.9	1.03 (0.75–1.42)	*P*=0.852
24∼26.9	1.29 (0.91–1.81)	*P*=0.151
27∼29.9	1.33 (0.91–1.94)	*P*=0.143
>30	1.23 (0.80–1.89)	*P*=0.350

Number of embryos transferred		*P* < 0.001
1		
2	1.76 (1.46–2.14)	*P* < 0.001

Day of embryo transferred		
D3		
D4	1.81 (1.01–3.25)	*P*=0.045
D5	2.41 (1.95–2.98)	*P* < 0.001
D6	1.36 (1.13–1.63)	*P*=0.001

**Table 4:** Multivariate analysis of frozen-thawed embryo on Day 4 of transplantation as control.

	OR (95% CI)	*P*
Day of embryo transferred		*P* < 0.001
D4		
D3	0.55 (0.31–0.99)	*P*=0.045
D5	1.33 (0.73–2.41)	*P*=0.358
D6	0.75 (0.42–1.35)	*P*=0.331

**Table 5:** Multivariate analysis of frozen-thawed embryo on Day 5 of transplantation as control.

	OR (95% CI)	*P*
Day of embryo transferred		*P* < 0.001
D5		
D3	0.42 (0.34–0.51)	*P* < 0.001
D4	0.76 (0.42–1.37)	*P*=0.358
D6	0.56 (0.46–0.69)	*P* < 0.001

## Data Availability

The datasets used and/or analyzed during the current study are available from the corresponding author upon reasonable request.
